# A case report of acute myocardial infarction with extremely severe thrombocytopenia after percutaneous coronary intervention

**DOI:** 10.1097/MD.0000000000035971

**Published:** 2023-11-10

**Authors:** Shuai Chen, Hongqi Wang, Bin Ning

**Affiliations:** a Cardiovascular Department, Fuyang People’s Hospital Affiliated to Anhui Medical University, Fuyang, Anhui, China.

**Keywords:** acute myocardial infarction, percutaneous coronary intervention, thrombocytopenia

## Abstract

**Introduction:**

The case report’s purpose is to remind doctors a rare complication named thrombocytopenia of antithrombotic drugs. As a result, severe bleeding or even life-threatening situations may be avoided.

**Patient concerns:**

A specific case of a patient with acute myocardial infarction, a significant decrease in platelet count was observed after percutaneous coronary intervention.

**Diagnosis:**

After ruling out other potential causes, the medical team considered tirofiban-induced thrombocytopenia as a possible explanation.

**Interventions and outcomes:**

Through careful monitoring and adjustment of medication, the patient’s platelet count eventually returned to normal.

**Conclusion:**

To ensure patient safety, it is advised to regularly monitor platelet counts at intervals of 2 to 6 hours before and after administering tirofiban.

## 1. Introduction

The advancement of technology has led to the widespread use of coronary angiography and percutaneous coronary intervention (PCI) in the evaluation of patients with coronary artery disease. Glycoprotein (GP) IIb/IIIa receptor antagonists, known as platelet antiaggregation agents, are being used more frequently in the management of acute coronary syndromes and post PCI. Although thrombocytopenia is a common complication, it is rare in this particular treatment category. In this report, we present a case of acute coronary syndromes patients who underwent PCI and experienced extremely severe thrombocytopenia following tirofiban administration.

## 2. Patient information

The 65-year-old male patient was admitted to the hospital with a complaint of chest pain that had been present for 3 hours. At 14:10 on the same day, the patient experienced sudden onset of persistent pressure pain on the lower part of the sternum, with no apparent cause. The patient also reported symptoms of sweating and suffocation, but did not experience radiation of pain to the shoulder or back, dizziness, amenorrhea, syncope, cough, hemoptysis, dyspnea, laceration-like pain in the back, abdominal pain, diarrhea, nausea, vomiting, hematemesis, melena, waist soreness, back pain, or fear of chills, shivering, and fever. At 15:14, the electrocardiogram (ECG) indicated acute inferior wall muscle infarction. The patient received a pack of oral drugs with a loading dose and was recommended to transfer to a superior hospital for treatment. He contacted 120 for the transfer and arrived at our hospital’s emergency department at 16:00. At 16:05, the ECG showed sinus rhythm with a heart rate of 61 bpm. There was ST-segment elevation of approximately 0.2 mV in leads II, III, and aVF, and ST-segment depression of 0.1 to 0.3 mV in leads I, aVL, V1-V5. The patient continued to experience chest pain. At 16:06, the emergency department called our department for consultation, and at 16:07, the chief resident arrived at the emergency department. The patient was diagnosed with acute inferior myocardial infarction and had indications for emergency PCI. At 16:11, the catheterization laboratory was initiated, and the patient was recommended to undergo emergency PCI. After careful consideration, the patient and their family agreed to proceed with the operation. Angiography commenced at 16:41 and concluded at 16:44, revealing a complete occlusion of the middle segment of the right coronary artery with thrombus shadow. At 16:47, the guide wire was successfully inserted, followed by the use of a suction catheter for coronary aspiration. Upon reexamination, diffuse lesions were observed in the middle segment of the coronary artery, with residual stenosis of approximately 90%. The operation was successfully completed, and the patient was accompanied by medical staff to our department’s ward. Throughout the course of the disease, the patient remained alert and exhibited high energy levels. The patient reported normal diet, sleep, urination, and defecation. The past medical history included a 19-year history of hepatitis B carrier, while the rest of the medical history was unremarkable.

## 3. Clinical findings

**Figure 1.**
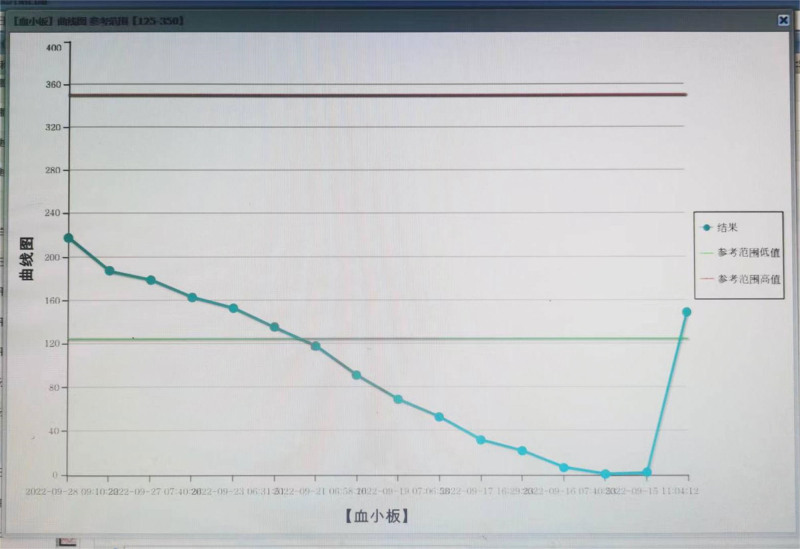
Line chart illustrating changes in platelet count.

The physical examination revealed a blood pressure of 137/85 mmHg, good spirit, stable breathing, no cyanosis of the lips, no jugular vein distention, clear breath sounds in both lungs without any dry or wet rales, normal heart size with a heart rate of 70 bpm and regular rhythm, no murmurs in the valve area, a soft abdomen, liver and spleen not palpable below the ribs, no swelling in the lower limbs, and a normal nervous system. The ECG of the emergency department at our hospital showed sinus rhythm at a rate of 61 bpm. The ST segment height in leads II, II, and aVF was approximately 0.2 mV, while the ST segment depression in leads I, aVL, V1-V5 ranged from 0.1 to 0.3 mV. The postoperative ECG revealed sinus rhythm at a rate of 99 bpm, with a Q wave in leads II, III, and AVF, and the ST segment in each lead returned to the baseline level. The electrocardiogram before and after the emergency operation are depicted in Figure 1.

## 4. Diagnostic assessment

The initial diagnosis was acute ST-segment elevation myocardial infarction, Killip grade I, coronary atherosclerotic heart disease.

## 5. Therapeutic intervention

Following the operation, the patient was prescribed aspirin 100 mg qd, ticagrelor 90 mg bid, rosuvastatin calcium 10 mg qn, and tirofiban hydrochloride 1.4 mL/hour (temporary), along with famotidine.

## 6. Follow-up and outcomes

On the second day post-operation, the laboratory department reported a critically low platelet count of 2 × 10^9^/L. To ensure accuracy, a blood routine reexamination revealed a platelet count of 1 × 10^9^/L and no platelets were observed under microscopy, thus confirming the absence of laboratory errors. As a result, the administration of aspirin, ticagrelor, and tirofiban was immediately ceased. Following consultation with the hematology department, the possibility of heparin-induced thrombocytopenia (HIT) could not be ruled out at that time, and platelet transfusion was not deemed necessary. Close monitoring of routine blood tests, as well as skin and mucosal bleeding, was implemented.

**Figure 2.**
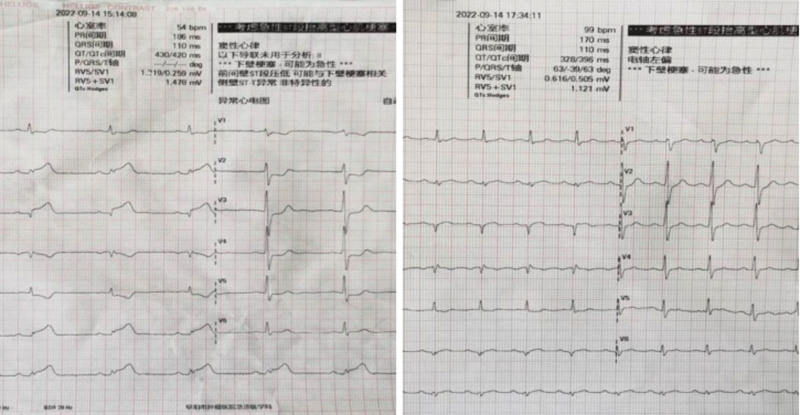
The ECG before and after emergency surgery. ECG = electrocardiogram.

Following drug withdrawal, the platelet count showed a progressive increase. One day after drug withdrawal, the platelet count increased to 7 × 10^9^/L, followed by further increases to 22 × 10^9^/L on the second day, 53 × 10^9^/L on the third day, and 69 × 10^9^/L on the fourth day. At this point, ticagrelor 90 mg bid was administered. After 6 days of drug withdrawal, the platelet count reached 118 × 10^9^/L, and ticagrelor was combined with aspirin (100 mg qd). The changes in platelet count can be observed in Figure 2.

Color Doppler echocardiography was performed, indicating segmental ventricular wall motion abnormality. A bedside chest X-ray revealed slightly enhanced texture in both lungs. A follow-up coronary angiography on September 27, 2020 showed that initially, the trend of the coronary artery was normal. However, there was plaque infiltration in the distal segment of the left main coronary artery without significant stenosis. There was no apparent stenosis in the proximal segment of the anterior descending coronary artery. In the middle segment, there were diffuse long lesions, with the most severe stenosis being approximately 70%. Plaque shadow was observed, but no significant stenosis was found in the distal segment. The proximal segment of the left circumflex artery showed about 50% stenosis. Local dilatation was observed after the stenosis, and the distal segment of the artery had an irregular intima but no significant stenosis. The proximal segment of the right coronary artery had plaque infiltration but no significant stenosis. The middle segment had a long lesion with the most severe stenosis, approximately 90%. The distal segment of the artery also had an irregular intima but no significant stenosis. After considering all factors, it was recommended to treat the culprit vessel, the right coronary artery, with the implantation of 2 stents. The operation was successful, and post-operation, anti-platelet aggregation and lipid-lowering treatments were continued to stabilize the plaque. Following discharge, the patient was prescribed aspirin 100 mg once daily, ticagrelor 90 mg twice daily, rosuvastatin calcium tablets 10 mg every night, metoprolol tartrate tablets 12.5 mg twice daily, and rabeprazole 10mg once daily. Due to the patient’s low blood pressure, angiotensin receptor blocker drugs were temporarily not administered.

## 7. Discussion

Mild thrombocytopenia was defined as a platelet count less than 100 × 10^9^/L, severe thrombocytopenia was defined as a platelet count less than 50 × 10^9^/L, and extremely severe thrombocytopenia was defined as a platelet count less than 20 × 10^9^/L within 24 hours of tirofiban application. In this case, the patient had received multiple antithrombotic agents, such as aspirin, ticagrelor, unfractionated heparin, and tirofiban, prior to developing thrombocytopenia. The clinical diagnostic criteria for tirofiban-induced thrombocytopenia mainly rely on the relationship between the timing of tirofiban administration and the occurrence of thrombocytopenia, while other possible causes need to be ruled out.

**Figure 3.**
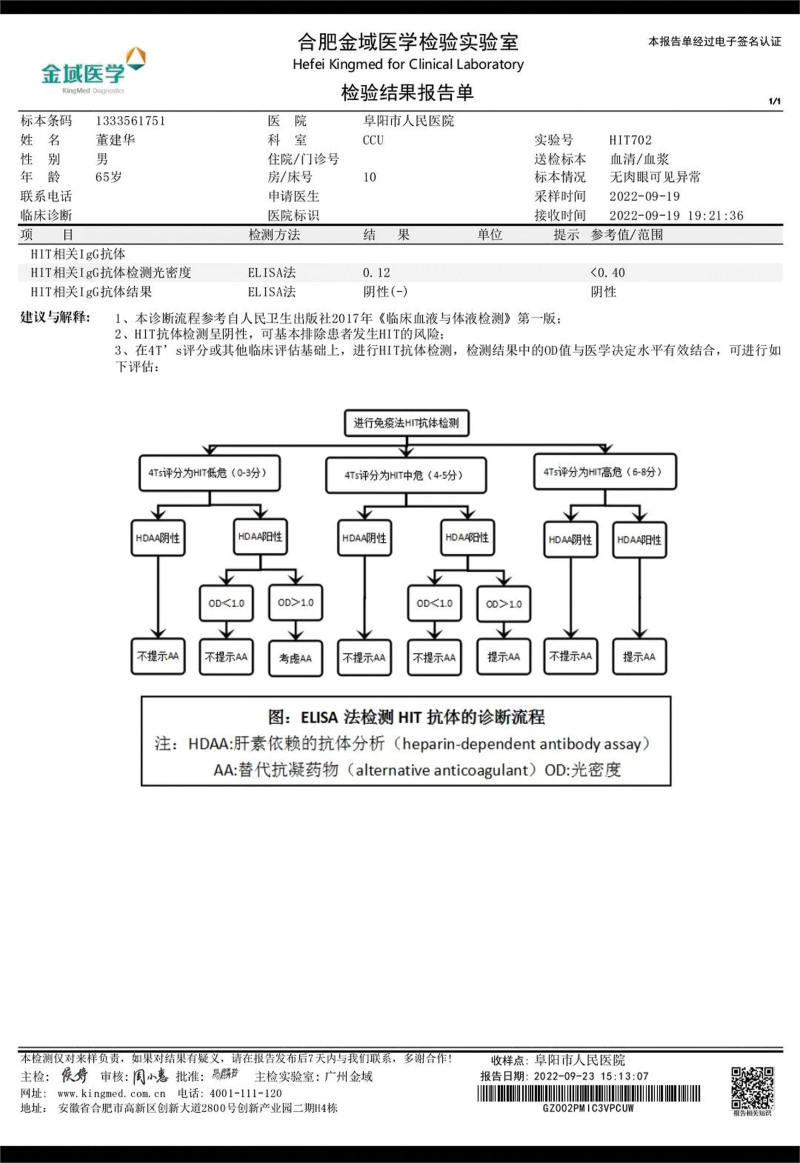
The HIT test results of the patient. HIT = heparin-induced thrombocytopenia.

The main differential diagnosis for thrombocytopenia in this patient is HIT. HIT is caused by autoantibodies to platelet factor 4 (PF4), which forms a complex with heparin. This complex activates platelets and leads to extensive arterial and venous thrombosis, as well as thrombocytopenia. HIT is classified into 2 types. Type I HIT occurs within 2 days of heparin exposure and is characterized by a decrease in platelet count of at least 50 × 10^9^/L. Type II HIT typically occurs 5 to 14 days after heparin exposure in patients who have not received heparin before. In this case, the patient had no previous exposure to heparin and developed early and severe thrombocytopenia that did not fit the typical features of type I or II HIT. The “4Ts score”^[1]^ is a scoring system used to diagnose HIT in a standardized manner. In this case, the patient has a 4T’s score of 1, indicating a low likelihood of disease onset (<5% probability). The severity of thrombocytopenia was scored as 0 (minimum value < 10 × 10^9^/L), the timing of thrombocytopenia after heparin use was scored as 0 (heparin use < 5 days, no recent heparin use), the presence of thrombosis was scored as 0 (none), and other potential causes of thrombocytopenia were scored as 1 (possible). The negative HIT antibody test effectively ruled out HIT in this patient. The test results of HIT antibody are illustrated in Figure 3.

Dual antiplatelet medication is a possible cause of thrombocytopenia. However, dual antiplatelet medication-induced thrombocytopenia usually takes days or weeks to develop, unlike the rapid onset observed in this patient. Furthermore, the patient’s platelet count returned to the normal range after discontinuation of tirofiban. Therefore, it is likely that tirofiban caused the thrombocytopenia in this case. To confirm this hypothesis, a case^[2]^ reported in 2007 utilized ELISA and flow cytometry to demonstrate that drug-dependent antibodies were formed only in the presence of tirofiban. The patient had normal platelet levels prior to surgery, but experienced a significant drop in platelet count, known as thrombocytopenia, after the surgical procedure. The possibility of drug-induced immune thrombocytopenia was taken into consideration. Drug-induced immune thrombocytopenia is a condition where platelet destruction is caused by antibodies that are dependent on the presence of certain drugs. It is important for patients to avoid the specific drug indefinitely in the future, as drug-dependent antibodies can persist for extended periods of time.^[3]^

GP IIb/IIIa receptor antagonists belong to a class of antiplatelet drugs that work by reducing platelet aggregation and the formation of blood clots.^[4]^ It is increasingly being utilized for the treatment of acute coronary syndromes and as a follow-up after PCI.^[5]^ These drugs block specific binding sites that are necessary for platelet aggregation. Tirofiban, for example, is a reversible and competitive inhibitor of the GP IIb/IIIa receptor. It recognizes the Arg-Gly-Asp sequence in the β3 subunit of the receptor, ultimately leading to the inhibition of blood clot formation.^[6]^ GP IIb/IIIa antagonists are commonly used in patients with acute coronary syndromes and those undergoing coronary stent implantation. They are effective in preventing platelet aggregation and the formation of blood clots. It has been reported that GP IIb/IIIa receptor antagonists can cause diffuse alveolar hemorrhage,^[7]^ which can be life-threatening. Salma Abdeladim et al^[8]^ reported 2 patients who experienced extremely severe thrombocytopenia and massive bleeding after emergency coronary intervention with tirofiban and unfractionated heparin. Amit Gulati et al^[9]^also reported a case of thrombocytopenia with platelet count dropping to 4 × 10^9^/L within 1 day after tirofiban administration. In a separate study conducted by Christen et al,^[10]^ a patient who had inferior wall myocardial infarction and underwent PCI experienced thrombocytopenia as a result of tirofiban. This patient later developed shortness of breath and was subsequently diagnosed with diffuse alveolar hemorrhage. In this particular case, the patient developed extremely severe thrombocytopenia, but no adverse events such as bleeding occurred, mainly due to the timely discovery and discontinuation of antiplatelet aggregation drugs. It is recommended to monitor platelet counts at 2 to 6 hour intervals before and after tirofiban administration.

Tirofiban is cleared from the circulation within 1 hour of discontinuation.^[11]^ After discontinuation, platelet counts begin to recover, and treatment options such as steroids, Ig-G immune globulin, and platelet transfusions can be considered. However, platelet transfusions are not recommended for patients with severe thrombocytopenia without overt bleeding due to the potential risk of stent thrombosis or reinfarction. It is important to exercise caution when using Thrombopoietin in the treatment of glycoprotein IIb/IIIa receptor antagonists introduced thrombocytopenia, considering the possible risk of thrombosis.^[12]^

## 8. Limitations

The limitation of this study is that tirofiban-induced thrombocytopenia is a diagnosis made by excluding other possibilities such as HIT, aspirin, and GPIIb/IIIa inhibitors.

## Author contributions

**Writing—original draft:** Shuai Chen.

**Writing—review & editing:** Hongqi Wang, Bin Ning.
